# Pembrolizumab‐Induced Severe Pneumonitis in a Patient With Lung Adenocarcinoma After Umbilical Cord Blood Transplantation: A Case Report

**DOI:** 10.1002/rcr2.70307

**Published:** 2025-08-04

**Authors:** Shunta Yamamoto, Hiroki Kabata, Yuri Fukasawa, Kohei Fukuzawa, Masahito Mizobe, Risako Suzuki, Akira Miyakawa, Keiko Ohgino, Hideki Terai, Jun Kato, Hiroyuki Yasuda, Koichi Fukunaga

**Affiliations:** ^1^ Division of Pulmonary Medicine, Department of Medicine Keio University School of Medicine Tokyo Japan; ^2^ Division of Hematology, Department of Medicine Keio University School of Medicine Tokyo Japan

**Keywords:** immune checkpoint inhibitors, irAE, lung cancer, PD‐1, secondary malignancies

## Abstract

Immune checkpoint inhibitors (ICIs) are essential treatments for lung cancer, but their safety following allogeneic haematopoietic stem cell transplantation (allo‐HSCT) remains unclear. We report a case of a 66‐year‐old man who underwent umbilical cord blood transplantation for acute lymphoblastic leukaemia and later developed lung adenocarcinoma. Pembrolizumab monotherapy was initiated, but the patient developed severe steroid‐refractory immune‐related pneumonitis, leading to mortality 56 days after treatment initiation. Given the scarcity of reports on ICI use in lung cancer patients with prior HSCT, this case highlights critical safety considerations.

## Graphical abstract

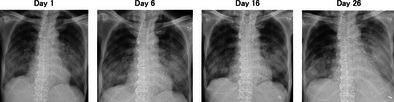

## 1 Introduction

Recent advancements in cancer treatment have significantly improved survival rates, leading to a growing population of cancer survivors. Immune checkpoint inhibitors (ICIs) play a crucial role in lung cancer treatment but are associated with immune‐related adverse events (irAEs). However, their safety and efficacy in patients with a history of allogeneic haematopoietic stem cell transplantation (allo‐HSCT) and subsequent lung cancer remain unclear. Here, we report the case of a 66‐year‐old man with lung adenocarcinoma, treated with pembrolizumab, who had undergone umbilical cord blood transplantation for acute lymphoblastic leukaemia.

## 2 Case Report

**FIGURE 1:**
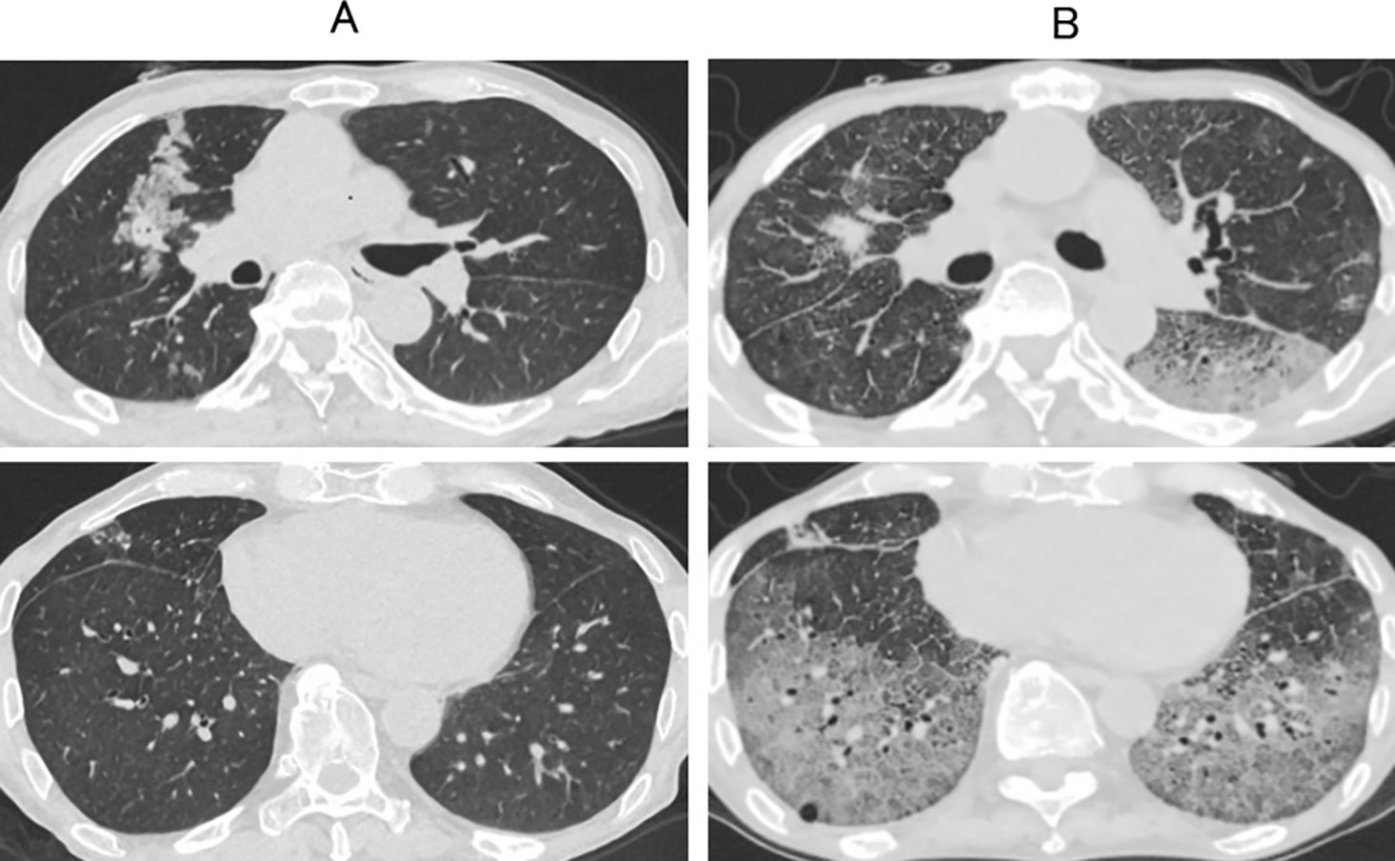
(A) Chest CT scan before pembrolizumab initiation. (B) Chest CT scan at the time of hospitalisation.

A 66‐year‐old man with a history of Philadelphia chromosome‐positive acute lymphoblastic leukaemia underwent umbilical cord blood transplantation in February of the previous year. Subsequently, a nodule developed within a pulmonary cyst, and aspergilloma was suspected, leading to regular follow‐up. In April of this year, a chest x‐ray revealed progressive enlargement of the nodule in the right middle lobe. PET‐CT demonstrated high FDG avidity in the nodule (SUVmax 8.9), as well as ipsilateral hilar lymph node metastasis and bone metastases. A CT‐guided biopsy confirmed a diagnosis of lung adenocarcinoma (cT1bN1M1c, stage IVB), with no detectable driver mutations and a PD‐L1 tumour proportion score of 70%. Given concerns about susceptibility to infections due to chemotherapy, pembrolizumab monotherapy was initiated on May 9 based on the KEYNOTE‐024 trial [1]. However, on day 27 of treatment, the patient experienced dyspnea and loss of consciousness at home, prompting emergency transport to the hospital (Figure 1).

**FIGURE 2:**
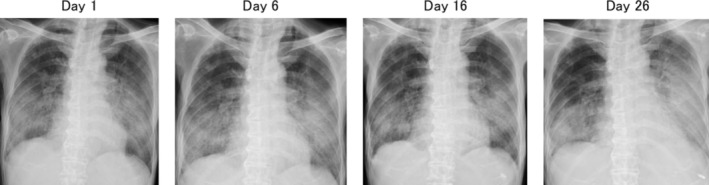
(Day 1) Chest x‐ray at the time of hospitalisation. (Day 6) Chest x‐ray after initiation of high‐dose corticosteroid therapy. (Day 16) Chest x‐ray after initiation of cyclophosphamide. (Day 26) Chest x‐ray after initiation of ruxolitinib.

On admission, his vital signs were as follows: temperature, 37.2°C; blood pressure, 138/83 mmHg; heart rate, 106 bpm; and SpO_2_, 92% with 8 L/min oxygen via mask. Laboratory findings showed elevated white blood cell count, LDH, KL‐6, and CRP. However, CMV antigen, Aspergillus antigen, and β‐D‐glucan tests were negative, as were SARS‐CoV‐2 and influenza PCR tests. Based on these findings, infectious causes were considered unlikely. Given the early onset of symptoms after initiation of pembrolizumab monotherapy, along with elevated LDH and KL‐6, the patient was clinically diagnosed with irAE pneumonitis. Upon hospitalisation, high‐dose corticosteroid therapy (methylprednisolone 1000 mg/day for 3 days) was initiated alongside high‐flow nasal cannula (HFNC) oxygen therapy. In addition, empirical antimicrobial therapy with piperacillin/tazobactam (PIPC/TAZ) and azithromycin (AZM) was initiated. Despite repeated cultures and blood tests after admission, no evidence of infection was found. Bronchoscopy was not performed due to the patient's poor general condition. Although a temporary decrease in LDH levels and improvement in chest x‐ray findings were observed, oxygenation did not improve. On day 11 of hospitalisation (day 38 of treatment), cyclophosphamide was administered, followed by intravenous immunoglobulin (IVIG) on day 16 (day 43 of treatment) and day 23 (day 50 of treatment). Additionally, on day 20 of hospitalisation (day 47 of treatment), a second course of high‐dose corticosteroids was given, and on day 22 (day 49 of treatment), ruxolitinib was initiated. However, respiratory function continued to deteriorate, and the patient suffered cardiac arrest on day 29 of hospitalisation (day 56 of treatment) (Figure 2).

## 3 Discussion

This case report presents a patient who developed lung cancer after allo‐HSCT and subsequently died due to steroid‐resistant irAEs following ICI treatment. Advances in cancer treatment have significantly improved the prognosis of cancer patients, resulting in an increase in long‐term survivors. However, as exemplified by this case, the incidence of secondary malignancies has also increased, possibly due to prolonged survival or the late effects of prior anti‐cancer treatment. In such cases, treatment decisions must take into account the impact of prior therapies.

After allo‐HSCT, donor‐derived T cells are continuously exposed to antigenic stimulation, leading to increased expression of PD‐1 and T cell exhaustion, which raises the risk of tumour development [2]. Therefore, the activation of antitumour immunity through ICIs offers substantial therapeutic benefits. Indeed, recent studies have demonstrated the efficacy of ICIs in relapsed haematologic malignancies after allo‐HSCT. However, the use of ICIs also increases the risk of irAEs and Graft‐Versus‐Host Disease (GVHD) [3]. For example, pembrolizumab induced grade 3–4 irAEs in 3 of 12 patients (25%) after allo‐HSCT [4]. Patients with a history of GVHD or early ICI administration after transplantation have an increased risk of GVHD, which decreases when ICIs are given > 180 days post‐transplant [4]. While ICIs provide substantial benefits in enhancing antitumour immunity, their use after HSCT is associated with the risk of both irAEs and GVHD, presenting a challenging clinical dilemma.

Approximately 1.6% of patients who received ICI treatment had irAE‐related pneumonia requiring second‐line immunosuppressants [5]. Secondary treatments such as mycophenolate mofetil, infliximab, IVIG, cyclophosphamide, and tocilizumab have been proposed, though their efficacy has not been adequately established [5]. In this case, high‐dose corticosteroids, IVIG, and cyclophosphamide were administered without significant improvement. Since both irAE and GVHD are considered to involve the activation of similar lymphocyte populations, ruxolitinib, a JAK1/2 inhibitor with established efficacy in GVHD [6], was also administered. Recent case reports have suggested that ruxolitinib may be effective for other steroid‐refractory irAEs, such as myocarditis and dermatitis [7, 8]. However, in this case, no clinical improvement was observed.

This case is the first report on the use of ICIs in lung cancer patients following allo‐HSCT, and further studies are necessary to establish effective treatment protocols. Further studies should clarify optimal timing and treatment algorithms to minimise GVHD and irAE risk following ICI administration. In addition, identifying biomarkers to predict the risk of irAEs represents an important area of future research. This case highlights the need for careful, individualised evaluation of ICI treatment in lung cancer patients after allo‐HSCT, balancing potential risks and benefits to optimise patient outcomes.

## Author Contributions

**Shunta Yamamoto:** writing – original draft. **Hiroki Kabata:** writing – review and editing, project administration. **Yuri Fukasawa:** resources. **Kohei Fukuzawa:** resources. **Masahito Mizobe:** resources. **Risako Suzuki:** resources. **Akira Miyakawa:** resources. **Keiko Ohgino:** resources. **Hideki Terai:** resources. **Jun Kato:** writing – review and editing, resources. **Hiroyuki Yasuda:** supervision. **Koichi Fukunaga:** supervision.

## Consent

The authors declare that written informed consent was obtained for the publication of this manuscript and accompanying images using the form provided by the Journal.

## Conflicts of Interest

The authors declare no conflicts of interest.

## Data Availability

Data sharing not applicable to this article as no datasets were generated or analysed during the current study.

## References

[1] M. Reck , D. Rodríguez‐Abreu , A. G. Robinson , et al., “Pembrolizumab Versus Chemotherapy for PD‐L1‐Positive Non‐Small‐Cell Lung Cancer,” New England Journal of Medicine 375, no. 19 (2016): 1823–1833. 27718847 10.1056/NEJMoa1606774

[2] S. Asakura , D. Hashimoto , S. Takashima , et al., “Alloantigen Expression on Non‐Hematopoietic Cells Reduces Graft‐Versus‐Leukemia Effects in Mice,” Journal of Clinical Investigation 120 (2010): 2370–2378. 20530875 10.1172/JCI39165 PMC2898583

[3] S. H. Baumeister , G. J. Freeman , G. Dranoff , and A. H. Sharpe , “Coinhibitory Pathways in Immunotherapy for Cancer,” Annual Review of Immunology 34 (2016): 539–573. 10.1146/annurev-immunol-032414-112049 26927206

[4] J. Godfrey , H. Liu , J. Yu , et al., “Pembrolizumab for the Treatment of Disease Relapse After Allogeneic Hematopoietic Stem Cell Transplantation,” Blood Advances 7 (2023): 963–970. 35973200 10.1182/bloodadvances.2022008403 PMC10027501

[5] S. Ogusu , Y. Harutani , T. Tozuka , et al., “Second‐Line Immunosuppressant Administration for Steroid‐Refractory Immune‐Related Adverse Events in Patients With Lung Cancer,” Cancer Immunology, Immunotherapy 72 (2023): 3765–3772. 37638979 10.1007/s00262-023-03528-x PMC10576678

[6] R. Zeiser , N. Polverelli , R. Ram , et al., “Ruxolitinib for Glucocorticoid‐Refractory Chronic Graft‐Versus‐Host Disease,” New England Journal of Medicine 385 (2021): 228–238. 34260836 10.1056/NEJMoa2033122

[7] E. Wadden , C. Lai , P. Grivas , et al., “Successful Treatment of Immune Checkpoint Inhibitor‐Associated Fulminant Myocarditis With Abatacept and Ruxolitinib: A Case Report,” European Heart Journal ‐ Case Reports 9, no. 2 (2025): ytaf019. 39963309 10.1093/ehjcr/ytaf019 PMC11831032

[8] C. M. Powers , H. Verma , J. Orloff , et al., “Use of a Topical Janus Kinase Inhibitor in Immune Checkpoint Inhibitor‐Induced Eczematous Reaction: A Case Report,” Journal of Dermatological Treatment 35 (2024): 2336118. 38565207 10.1080/09546634.2024.2336118

